# GLP-1 receptor agonists in obesity-related knee osteoarthritis: from weight loss to therapeutic pathway reconstruction

**DOI:** 10.3389/fphar.2026.1856307

**Published:** 2026-06-17

**Authors:** Jincan Wu, Ying Lei, Ren Sa, Yongquan Zhong, Yunqiong Lu

**Affiliations:** 1 Department of Massage Therapy, Xiamen Hospital of Traditional Chinese Medicine, Xiamen, China; 2 Department of Acupuncture, Sanya Hospital of Traditional Chinese Medicine, Sanya, China

**Keywords:** glucagon-like peptide-1 receptor agonists, knee osteoarthritis, obesity, treatment pathway, weight loss

## Abstract

Obesity is a major modifiable contributor to knee osteoarthritis (KOA), but excess adiposity has often been managed as a background risk factor rather than as a therapeutic target. In obesity-related KOA, adiposity may aggravate pain and disability through increased joint loading, low-grade inflammation, metabolic dysfunction, impaired physical activity, and reduced rehabilitation tolerance. Glucagon-like peptide-1 receptor agonists (GLP-1RAs) have created a new opportunity to achieve clinically meaningful weight loss in selected patients with obesity-related KOA. However, current evidence should be interpreted cautiously. Human data most strongly support weight-loss-mediated improvements in pain, function, and rehabilitation feasibility, whereas direct cartilage-, synovium-, subchondral bone-, or structure-modifying effects remain unproven. In this narrative review, we summarize the rationale, receptor biology, pharmacological mechanisms, inflammatory signaling hypotheses, clinical evidence, safety concerns, and implementation challenges related to GLP-1RA use in obesity-related KOA. We also position GLP-1RAs within a broader obesity-treatment continuum that includes lifestyle intervention, exercise-based rehabilitation, multidisciplinary weight management, other anti-obesity medications, bariatric surgery, symptom-bridging treatments, and arthroplasty when indicated. Particular attention is given to gastrointestinal intolerance, dehydration, gallbladder and pancreatitis-related concerns, lean-mass loss, perioperative management, treatment discontinuation, affordability, access, and long-term adherence. Overall, GLP-1RAs may become a useful component of integrated obesity-directed KOA care, but they should be regarded as part of a proposed framework rather than as validated disease-modifying therapy.

## 1 Introduction

Knee osteoarthritis (KOA) is a major cause of chronic pain, physical limitation, and reduced quality of life in middle-aged and older adults. Although both pharmacological and nonpharmacological interventions can improve symptoms and function, current treatment remains largely palliative and does not fundamentally reverse the pathological or radiographic course of disease. Among the modifiable risk factors for KOA, overweight and obesity are consistently regarded as the most important, influencing not only disease onset but also symptom burden, functional decline, and long-term therapeutic response (Gelber, 2024).

Importantly, obesity-related KOA should no longer be viewed as a purely mechanical disorder caused by excess joint loading. Accumulating evidence supports a more complex model in which obesity contributes to KOA through both biomechanical and systemic mechanisms. Excess body weight increases knee joint loading, alters gait mechanics, and accelerates degeneration of the osteochondral unit (Shumnalieva et al., 2023; Chen et al., 2020). At the same time, adipose tissue functions as an active endocrine and immunometabolic organ, promoting low-grade inflammation, adipokine dysregulation, oxidative stress, and metabolic disturbance, all of which may influence the synovium, cartilage, subchondral bone, ligaments, and periarticular muscle (Shumnalieva et al., 2023; Chen et al., 2020; Batushansky et al., 2022). This broader view is consistent with the modern concept of OA as a whole-joint disease rather than an isolated cartilage disorder (Shumnalieva et al., 2023; Batushansky et al., 2022).

From a clinical standpoint, obesity-related KOA also appears to represent a distinct and therapeutically challenging phenotype. Patients with concomitant obesity and metabolic dysfunction often have more severe pain, greater disability, and a heavier overall symptom burden. Prior work has shown that metabolic syndrome and its components are associated with worse osteoarthritis symptomatology, including pain and impaired knee function (Li et al., 2016). These observations suggest that obesity in KOA is not merely a background characteristic but a biologically and clinically relevant disease driver. As a result, weight control should not be treated as a generic lifestyle recommendation alone, but as a central component of disease management (Li et al., 2016; Huffman et al., 2024).

Current guidelines already place physical activity and weight management at the core of non-surgical KOA care, yet implementation remains difficult in real-world practice. Many patients remain insufficiently active and continue to live with overweight or obesity, while lifestyle intervention alone often fails to produce substantial and durable weight loss (Huffman et al., 2024). In parallel, modern obesity medicine has evolved toward a comprehensive long-term care model that includes behavioral intervention, nutritional treatment, physical activity, pharmacotherapy, and metabolic or bariatric procedures when appropriate (Elmaleh-Sachs et al., 2023). Within this context, the emergence of glucagon-like peptide-1 receptor agonists, particularly semaglutide, has introduced a potentially important shift in the management of obesity-related KOA. In the STEP 9 trial, once-weekly semaglutide in adults with obesity and moderate-to-severe KOA was associated with significantly greater weight loss and greater improvement in knee pain than placebo at 68 weeks (Bliddal et al., 2024).

These findings raise a broader clinical question: how should anti-obesity pharmacotherapy be integrated into conservative care, rehabilitation, local symptom-relieving procedures, perioperative optimization, and arthroplasty decision-making for selected patients with obesity-related KOA? This narrative review addresses this question while distinguishing established clinical evidence from hypothesis-generating mechanisms and proposed care models. The literature was searched in PubMed/MEDLINE, Embase, Web of Science, and Google Scholar from database inception to 10 March 2026, using terms related to knee osteoarthritis, obesity, GLP-1 receptor agonists, incretin-based therapies, GLP-1 receptor biology, pharmacology, molecular mechanisms, inflammatory signaling, NF-κB, NLRP3 inflammasome, macrophage polarization, weight loss, arthroplasty, perioperative management, sarcopenia, safety, and cost-effectiveness. Randomized trials, systematic reviews, meta-analyses, clinical guidelines, observational studies, and mechanistic or translational studies were prioritized when they directly informed obesity-related KOA, GLP-1RA therapy, rehabilitation, surgical optimization, safety, or implementation. During revision, additional references were incorporated only when they directly addressed the reviewers’ comments, including reviewer-suggested literature, and were available on or before 10 March 2026. As this was a narrative review, formal risk-of-bias assessment and quantitative synthesis were not performed.

## 2 Before the GLP-1 era: the traditional treatment pathway and its limitations

Before the emergence of GLP-1-based anti-obesity pharmacotherapy, obesity-related KOA was generally managed within the same stepwise framework used for KOA overall, although overweight or obesity was explicitly recognized as a modifiable therapeutic target. Major guidelines consistently placed patient education, structured exercise, and weight loss at the core of first-line management, particularly for patients with knee OA who were overweight or obese. Pharmacological and procedural escalation was then typically layered on top of this foundation, with topical NSAIDs prioritized for knee pain, oral NSAIDs considered when appropriate, and intra-articular corticosteroid injections used for persistent symptoms (Kolasinski et al., 2020; Bannuru et al., 2019; Arden et al., 2021). In other words, the pre-GLP-1 treatment model was built on a rational premise: symptom control should be anchored to exercise and weight reduction, while medications and injections served as adjuncts when conservative therapy was insufficient.

This framework was not without evidence. In the IDEA randomized clinical trial, intensive diet-induced weight loss combined with exercise led to a mean weight reduction of 11.4% over 18 months and produced greater improvement in pain and function than exercise alone in overweight and obese adults with KOA (Messier et al., 2013). The mechanistic relevance of weight reduction was also supported by biomechanical work showing that each pound of weight lost was associated with a 4-fold reduction in knee joint load per step during daily activity (Messier et al., 2005). These findings reinforced the long-standing concept that weight loss is not merely a general health recommendation in KOA, but a disease-relevant intervention with the potential to reduce both symptom burden and mechanical stress across the joint (Messier et al., 2013; Messier et al., 2005).

However, the main weakness of the pre-GLP-1 pathway was not conceptual but operational. Weight loss was recommended early and repeatedly, yet sustained implementation was often difficult in the very population expected to benefit most. Knee pain, reduced mobility, deconditioning, and behavioral barriers all make weight reduction harder to achieve in patients with established KOA, creating a self-perpetuating cycle in which obesity worsens pain and disability, while pain and disability in turn undermine physical activity and long-term weight control (Wluka et al., 2013). As a result, the practical center of care often shifted away from disease-driver modification and toward symptom-directed escalation. In routine practice, many patients progressed from education and lifestyle advice to repeated use of analgesics, topical and oral NSAIDs, or injections, even though these approaches do not directly address obesity as a central driver of mechanical and metabolic disease burden (Kolasinski et al., 2020; Bannuru et al., 2019; Arden et al., 2021; Wluka et al., 2013).

This limitation became even more evident when patients approached end-stage disease and total knee arthroplasty (TKA). For many individuals with severe obesity, the pre-GLP-1 model created a therapeutic bottleneck: weight loss was frequently expected before surgery, but structured and effective weight-loss pathways were often lacking. In an observational study of patients denied TKA because of an institutional BMI cutoff, only 19.1% reached a BMI below 40 kg/m^2^ within 2 years in the absence of dedicated weight-loss protocols, whereas 53.1% neither underwent TKA nor became eligible during follow-up (Wilson et al., 2022). At the same time, obesity complicated rather than nullified surgical decision-making. A systematic review and meta-analysis found that morbidly obese patients experienced meaningful functional improvement after TKA, although they also had a higher overall burden of complications and a signal toward greater revision risk than non-obese patients (van Tilburg and Rathsach Andersen, 2022). Taken together, the pre-GLP-1 treatment pathway for obesity-related KOA was marked by a central paradox: weight reduction was considered fundamental, but was difficult to achieve and sustain; symptom-relieving therapies were readily available, but were largely non–driver-modifying; and surgery remained effective, yet was sometimes delayed or constrained by obesity-related risk and access barriers. This unresolved gap created the clinical context in which anti-obesity pharmacotherapy would later assume a potentially pathway-changing role.

## 3 Why anti-obesity pharmacotherapy may change the logic of KOA care

Modern anti-obesity pharmacotherapy may change the management of obesity-related KOA by making clinically meaningful weight reduction more achievable for selected patients. This is not a marginal difference. In a recent systematic review and meta-analysis of pharmacological treatments for obesity in adults, semaglutide and tirzepatide were among the agents associated with mean total body weight loss exceeding 10% versus placebo (McGowan et al., 2025). Against that background, the STEP 9 trial is especially important for KOA: among participants with obesity and moderate-to-severe knee pain, once-weekly semaglutide produced a mean body weight reduction of 13.7% versus 3.2% with placebo, together with a substantially greater improvement in WOMAC pain at 68 weeks (Bliddal et al., 2024). This magnitude of weight loss is clinically relevant because it may improve symptoms and function in selected patients with obesity-related KOA (Bliddal et al., 2024; McGowan et al., 2025).

For this reason, GLP-1-based therapy should not be interpreted simply as another add-on option for pain control. Rather, it supports a different clinical logic in which obesity itself is treated as a chronic, biologically active disease driver requiring active long-term management, not merely lifestyle advice repeated at each visit (Elmaleh-Sachs et al., 2023). This distinction is highly relevant in KOA, where older treatment pathways typically acknowledged the importance of weight reduction but relied heavily on exercise counseling, dietary advice, and later symptom-directed escalation, despite the well-recognized difficulty of achieving and maintaining substantial weight loss in routine care (Huffman et al., 2024; Elmaleh-Sachs et al., 2023). Within comprehensive obesity management, anti-obesity pharmacotherapy may therefore provide an additional option for selected patients who have difficulty achieving sufficient weight loss through lifestyle intervention alone (Elmaleh-Sachs et al., 2023; McGowan et al., 2025).

A second issue is mechanistic interpretation. The clinical benefit of GLP-1 receptor agonists in KOA should first be understood through a weight-loss-mediated pathway: substantial body-weight reduction may decrease knee joint loading, improve mobility, and make participation in exercise-based rehabilitation more feasible (Bliddal et al., 2024; Messier et al., 2005). Beyond this indirect pathway, GLP-1-based therapies may also improve systemic metabolic status and low-grade inflammation, which are biologically relevant to obesity-related OA. However, proposed cartilage-, synovium-, subchondral bone-, or intra-articular anti-inflammatory effects remain largely hypothesis-generating in humans, with stronger support from preclinical, translational, or indirect mechanistic evidence than from KOA-specific clinical trials (Ryan et al., 2025). Therefore, current evidence should be interpreted as supporting primarily weight-loss-associated symptomatic and functional benefit, rather than established direct joint-level disease modification (McGowan et al., 2025; Ryan et al., 2025). From a receptor pharmacology perspective, GLP-1 receptor agonists act through GLP-1R activation, a class B G-protein-coupled receptor signaling system with established systemic effects on appetite regulation, gastric emptying, glucose-dependent insulin secretion, metabolic control, and weight reduction (Drucker, 2025). In obesity-related KOA, these pharmacological effects may indirectly reduce mechanical joint loading and obesity-associated immunometabolic stress. Beyond this systemic pathway, preclinical and translational studies suggest several potential joint-relevant mechanisms, including activation of cAMP–PKA–CREB and PI3K–Akt signaling, attenuation of NF-κB and NLRP3 inflammasome pathways, modulation of macrophage polarization, reduction of endoplasmic-reticulum stress and oxidative stress, inhibition of AGEs–RAGE-related inflammatory signaling, and preservation of cartilage extracellular matrix homeostasis (Ryan et al., 2025; Drucker, 2025). These mechanisms provide biological plausibility for GLP-1RA-related effects on the obesity-related KOA joint microenvironment, but they remain hypothesis-generating in humans and should not be interpreted as proof of structural disease modification. This distinction is important because it allows GLP-1 receptor agonists to be discussed as a potential component of obesity-directed KOA care while avoiding the stronger, currently unproven claim that they directly modify joint structure or intra-articular disease activity in humans (Ryan et al., 2025).

**FIGURE 1:**
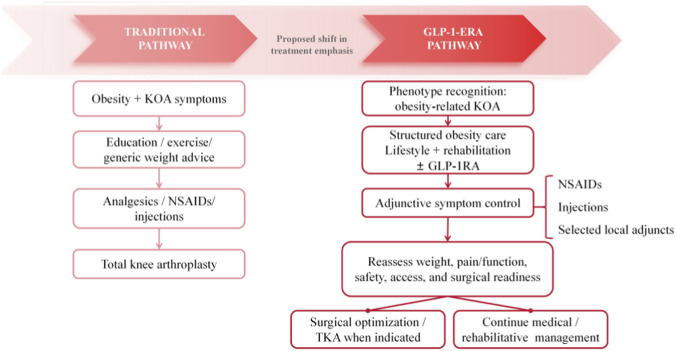
Proposed integration framework for GLP-1 receptor agonists in obesity-related knee osteoarthritis.

**FIGURE 2:**
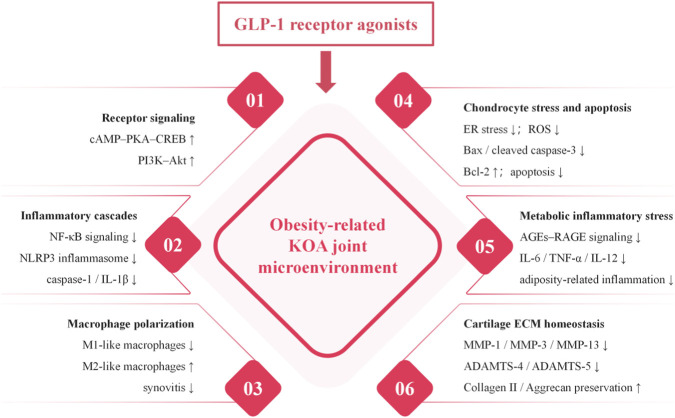
Proposed receptor pharmacology and inflammatory signaling mechanisms of GLP-1 receptor agonists in obesity-related knee osteoarthritis.

The broader drug-development landscape further supports this repositioning. In a recent overview of GLP-1-based therapeutics, Drucker highlighted that newer agents are producing greater and more rapid weight loss, may have important implications for musculoskeletal health, and are now being studied across indications extending beyond diabetes and obesity alone (Drucker, 2025). This shift is particularly visible in the TRIUMPH clinical development program, in which retatrutide is being evaluated not only for obesity but also for obesity-related complications including obstructive sleep apnea and knee osteoarthritis (Giblin et al., 2026). Notably, this program includes both nested OA protocols within basket-style obesity trials and a stand-alone phase 3 OA trial, indicating that knee OA is increasingly being treated as a clinically meaningful adiposity-related disease state in its own right (Giblin et al., 2026). Together, these developments suggest that GLP-1RAs may become part of a broader obesity-directed strategy for selected patients with KOA. However, their role should be interpreted as an emerging integration model rather than as a validated treatment pathway, particularly because direct KOA-specific evidence remains limited (Drucker, 2025; Giblin et al., 2026). This proposed integration model is summarized in Figure 1, whereas Figure 2 provides a focused mechanistic schematic of the proposed receptor pharmacology and inflammatory signaling pathways without duplicating the broader clinical integration framework shown in Figure 1. The key clinical, translational, perioperative, economic, and implementation-related evidence informing this proposed framework is summarized and critically appraised in Table 1, which also distinguishes direct human KOA evidence from indirect, observational, and hypothesis-generating evidence.

**TABLE 1:** Evidence map and critical appraisal of GLP-1 receptor agonists in obesity-related knee osteoarthritis.

Evidence source	Evidence status	Main finding	Main limitation	Reference
Bliddal et al.	Direct human KOA evidence	Semaglutide reduced body weight and improved knee pain and function in patients with obesity and KOA.	No structural imaging endpoint; follow-up was limited to 68 weeks; durability after stopping treatment remains unclear.	Bliddal et al. (2024)
McGowan et al.	Indirect obesity evidence	Modern anti-obesity drugs, especially semaglutide and tirzepatide, can produce substantial weight loss.	Not specific to KOA.	McGowan et al. (2025)
Ryan et al.	Mechanistic / hypothesis-generating evidence	GLP-1RAs may have anti-inflammatory and metabolic effects relevant to OA biology.	Mostly mechanistic or indirect evidence.	Ryan et al. (2025)
Cheng et al.	Preclinical and limited human evidence	Preclinical studies suggest possible cartilage, synovial, and inflammatory effects of GLP-1RAs.	Human KOA evidence remains limited.	Cheng et al. (2025)
Porto et al.	Observational pathway evidence	GLP-1RA exposure was associated with differences in OA-related treatment trajectories, including injections and arthroplasty.	Observational design; confounding and selection bias are likely.	Porto et al. (2025)
Lee et al.	Observational perioperative evidence	GLP-1RA use may be associated with better outcomes after joint arthroplasty.	Most included studies were retrospective.	Lee et al. (2025a)
Lee et al.	Observational perioperative meta-analysis	GLP-1RA use was associated with lower risks of some adverse outcomes after THA or TKA.	Residual confounding and healthier-user bias cannot be excluded.	Lee et al. (2025b)
Betensky et al.	Economic evidence	Semaglutide and tirzepatide may be cost-effective for patients with obesity and KOA.	Model-based results may not reflect real-world affordability.	Betensky et al. (2025)
Thomsen et al.	Real-world implementation evidence	Real-world weight loss may be lower than trial results, and gastrointestinal adverse effects often affect adherence.	Not specific to KOA.	Thomsen et al. (2025)
Singh et al.	Direct KOA systematic review	GLP-1RAs showed potential efficacy and safety signals for KOA management, but the available KOA-specific evidence remains limited.	Only a small number of direct studies were available; long-term safety, durability, and structural effects remain uncertain.	Singh et al. (2025)

Abbreviations: GLP-1RA, glucagon-like peptide-1 receptor agonist; KOA, knee osteoarthritis; OA, osteoarthritis; THA, total hip arthroplasty; TKA, total knee arthroplasty.

## 4 How GLP-1 receptor agonists may be integrated into obesity-related KOA care

### 4.1 From late rescue to earlier, phenotype-oriented intervention

In the traditional model, weight loss was recommended broadly but often functioned as a background aspiration rather than a realistic therapeutic milestone. The newer evidence base suggests that this position may no longer be adequate. A systematic review and meta-analysis of diet-induced weight loss interventions in overweight or obese individuals with KOA found that weight loss, especially when combined with exercise, was associated with improvements in pain and function (Hall et al., 2019). In parallel, a network meta-analysis reported that greater weight reduction was associated with greater improvement in WOMAC pain, stiffness, and function, while also emphasizing that exercise remains necessary to preserve lean mass and reduce the risk of sarcopenia during weight loss (Panunzi et al., 2021). Taken together, these data support a more selective and phenotype-oriented strategy in which patients with obesity-driven KOA, particularly those with high BMI, metabolic dysfunction, and clear weight-sensitive symptom burden, may benefit from earlier escalation to structured obesity treatment rather than prolonged reliance on repeated symptomatic rescue (Hall et al., 2019; Panunzi et al., 2021).

### 4.2 GLP-1 therapy should complement, not replace, exercise-based rehabilitation

Importantly, repositioning anti-obesity pharmacotherapy earlier in the pathway should not be interpreted as downgrading exercise therapy. On the contrary, the most coherent clinical model is one in which GLP-1-based treatment and rehabilitation are synergistic. Exercise remains foundational for muscle strength, joint stability, aerobic capacity, and long-term functional independence in KOA. What the GLP-1 era may change is not the importance of exercise, but the feasibility of engaging in it. Weight reduction may lessen pain during ambulation, reduce load-related symptom flares, and make progressive strengthening or aerobic rehabilitation more tolerable. This is particularly relevant because real-world lifestyle interventions, even when effective, may yield modest average symptom changes when translated outside specialized trial settings. In the community-based randomized trial by Messier et al., diet and exercise led to a statistically significant but small difference in knee pain over 18 months, with uncertain clinical importance (Messier et al., 2022). Rather than arguing against lifestyle therapy, these findings suggest that some patients with obesity-related KOA may require additional weight-management support to participate effectively in rehabilitation. In this framework, GLP-1RA therapy is best viewed as a potential adjunct to rehabilitation, not a substitute for exercise-based care (Hall et al., 2019; Panunzi et al., 2021; Messier et al., 2022).

### 4.3 Repositioning local procedures and adjunctive local therapies as bridging strategies

The GLP-1 era also has implications for the role of local symptom-directed interventions. Intra-articular corticosteroid injections, topical or oral analgesics, and other local procedures remain clinically relevant, particularly for short-term pain relief and for patients with acute symptom exacerbation (Bensa et al., 2024). Beyond these approaches, noninvasive local adjuncts such as thermotherapy, far-infrared–based interventions, and selected photobiomodulation protocols also merit consideration within a bridging framework. Small clinical studies have reported symptomatic benefit with far-infrared–emitting plasters and heat-and-steam thermotherapy in knee osteoarthritis, while broader review-level evidence suggests that infrared-based modalities may reduce pain in musculoskeletal conditions overall (Bagnato et al., 2012; Ochiai et al., 2014). At the same time, the evidence remains heterogeneous. Earlier review-level data concluded that thermotherapy studies in knee osteoarthritis were limited and methodologically inconsistent, and at least one placebo-controlled trial of monochromatic infrared energy reported no meaningful benefit in body function, activity, participation, or quality of life (Brosseau et al., 2003). Related low-level laser or photobiomodulation approaches may improve pain and disability when appropriate wavelengths and doses are used, but efficacy appears to be protocol-dependent and should not be generalized across all infrared-based interventions (Stausholm et al., 2019). In obesity-related KOA, these modalities are therefore best positioned as adjunctive tools to provide temporary symptom relief, facilitate mobilization, and improve participation in exercise and weight-management programs, rather than as stand-alone or disease-modifying treatments (Huang et al., 2015).

### 4.4 Surgical decision-making: Potential optimization rather than validated pathway reconstruction

Perhaps the most clinically consequential change concerns the relationship between obesity treatment and total knee arthroplasty. The key question is not whether GLP-1-based therapy will eliminate the need for TKA, because many patients with advanced structural disease will still require surgery, but whether it can improve the route by which patients arrive there. Existing arthroplasty literature already supports the value of preoperative optimization in patients with obesity. A 2024 systematic review and meta-analysis comparing TKA with or without prior bariatric surgery found that bariatric surgery before TKA was associated with lower risks of 90-day venous thromboembolism, 90-day stroke, and 1-year periprosthetic fracture, while most other outcomes were similar between groups (Sattari et al., 2024). At the same time, a 2024 systematic review concluded that there is limited and insufficient high-quality evidence to determine the optimal timing of arthroplasty after bariatric surgery (de Ree et al., 2024). A 2025 review in The Journal of Bone and Joint Surgery further emphasized that patients with obesity can still achieve meaningful functional improvement, but require preoperative medical optimization, weight management, consideration of bariatric surgery and GLP-1 receptor agonists, and multidisciplinary perioperative planning (Koh et al., 2025). In addition, a 2025 study reported that preoperative BMI improvement was associated with fewer TKA complications among patients with extreme, but not severe, obesity (Spezia et al., 2025). Collectively, these data suggest that anti-obesity pharmacotherapy may have a role in preoperative optimization for selected patients with obesity-related KOA. However, current evidence is insufficient to conclude that GLP-1 receptor agonists independently improve arthroplasty outcomes or safely delay surgery, because most surgical-transition data are observational and remain vulnerable to confounding, patient selection bias, and healthier-user effects.

## 5 Toward a practical integration model in the GLP-1 era

### 5.1 From generic weight advice to structured obesity care

One of the clearest lessons from the pre-GLP-1 era is that recommending weight loss is not the same as delivering obesity care. A systematic review of osteoarthritis clinical practice guidelines found that most guidelines for knee and hip OA recommend weight loss for patients with overweight or obesity, but very few specify how weight management should be operationalized in practice; only two guidelines specified a target of at least 5% weight loss, only one addressed maintenance of lost weight, and none explicitly recommended prevention of weight gain (Lim et al., 2022). A second systematic review focusing on higher-quality hip and knee OA guidelines similarly showed strong consistency around education, exercise, and weight management as core therapies, but also highlighted low applicability as a persistent weakness of guideline design, suggesting that implementation remains a major gap rather than a solved problem (Gibbs et al., 2023). The 2023 EULAR update reinforces this point by framing core management as an individualized, multicomponent strategy that includes education, self-management, tailored exercise, healthy weight maintenance or weight loss, assistive strategies when appropriate, and behavior change techniques to improve adherence (Moseng et al., 2024). Taken together, these sources suggest that the main challenge in obesity-related KOA is no longer whether weight management should be recommended, but how it should be delivered in a sufficiently structured, durable, and personalized way to change the clinical course of disease.

### 5.2 Patient selection should be driven by complication burden, not BMI alone

This shift has important implications for patient selection. As Conrozier noted, despite the well-established link between obesity and OA, there have historically been no specific recommendations for the medical management of obese patients with OA beyond advising weight loss and regular physical activity in addition to usual care (Conrozier, 2020). In contrast, recent work in precision obesity medicine argues for a phenotype-guided, complication-oriented approach, in which pharmacologic therapy is not positioned merely as the next step after lifestyle failure, but is selected according to the biological and clinical burden of obesity-related complications across the life course (Tuccinardi et al., 2025). Within such a framework, osteoarthritis can be considered a mechanical complication of obesity that may justify earlier use of anti-obesity medication when excess adiposity is clearly contributing to pain, disability, reduced exercise tolerance, or difficulty accessing definitive treatment (Chu et al., 2018). This reasoning also argues against relying on BMI alone as the trigger for escalation. A complication-driven model is more consistent with the emerging view that some patients with substantial symptom burden, recurrent need for symptom-directed interventions, or imminent surgical decision-making may warrant earlier obesity treatment even when traditional thresholds do not fully capture their musculoskeletal risk.

### 5.3 Treatment targets should extend beyond pain relief alone

A practical GLP-1-era pathway for obesity-related KOA should also redefine what counts as treatment success. Pain reduction remains essential, but it is too narrow to serve as the sole endpoint in a condition shaped by adiposity, mobility limitation, metabolic risk, and surgical complexity. A meta-analysis of adults with knee OA and obesity found that a 5% to 10% weight loss significantly improved pain, self-reported disability, and physical quality of life (Chu et al., 2018). At the same time, the literature reviewed above suggests that larger weight loss is generally associated with greater symptom improvement, while preservation of muscle mass and physical function remains a critical concern during weight reduction. In practical terms, this means that treatment goals in obesity-related KOA should be multidimensional: clinically meaningful weight reduction, improved pain and walking tolerance, better participation in exercise-based rehabilitation, preservation of lean mass and strength, and, where relevant, improved readiness for surgery (Panunzi et al., 2021; Moseng et al., 2024; Ursini et al., 2025). Framing outcomes in this broader way may help prevent the common but incomplete interpretation of GLP-1 therapy as simply a “pain drug by another route,” when in fact its clinical value may lie in altering the conditions under which rehabilitation, local procedures, and arthroplasty are pursued.

### 5.4 Positioning GLP-1 receptor agonists among obesity-treatment options

GLP-1 receptor agonists should be positioned within the broader continuum of obesity treatment rather than presented as a replacement for existing strategies. Intensive lifestyle intervention remains foundational because it addresses diet quality, physical activity, muscle strength, and long-term self-management. In patients with obesity-related KOA, this foundation is especially important because exercise and resistance training are necessary not only for weight control, but also for preserving quadriceps strength, balance, walking capacity, and rehabilitation potential (Messier et al., 2013; Hall et al., 2019; Panunzi et al., 2021; Messier et al., 2022).

Multidisciplinary weight-management programs may be particularly useful for patients whose knee pain, reduced mobility, or metabolic comorbidities make self-directed lifestyle change difficult. Such programs can integrate dietary intervention, behavioral support, physical therapy, pain control, and medication review, thereby providing a more realistic pathway than repeated advice to “lose weight” alone (Elmaleh-Sachs et al., 2023; Lim et al., 2022; Gibbs et al., 2023; Moseng et al., 2024). In this context, GLP-1 receptor agonists may act as a pharmacologic facilitator of weight reduction and rehabilitation participation, but they should not replace structured exercise, nutritional support, or long-term behavioral care.

Bariatric surgery remains an important option for eligible patients with severe obesity, especially when substantial and durable weight loss is needed or when obesity-related comorbidities are prominent. However, its relationship with arthroplasty timing remains complex, and available evidence does not yet define an optimal interval between bariatric surgery and joint replacement (Sattari et al., 2024; de Ree et al., 2024). Therefore, bariatric surgery and GLP-1 receptor agonists should be viewed as potentially complementary obesity-treatment options rather than competing pathways. Treatment selection should consider BMI, obesity-related complications, KOA severity, surgical candidacy, patient preference, local expertise, cost, and expected adherence (Sattari et al., 2024; de Ree et al., 2024; Koh et al., 2025).

Other anti-obesity medications may also be considered according to efficacy, contraindications, tolerability, availability, and cost. Compared with older pharmacologic options, GLP-1 receptor agonists and newer incretin-based therapies generally offer greater average weight reduction, but they also raise specific issues related to gastrointestinal tolerability, long-term continuation, affordability, and access (McGowan et al., 2025). Accordingly, the most appropriate role of GLP-1 receptor agonists in obesity-related KOA is as one component of individualized obesity care, integrated with lifestyle intervention, rehabilitation, multidisciplinary weight management, bariatric approaches when appropriate, and shared decision-making.

## 6 Safety and practical prescribing considerations in KOA patients

Safety considerations are particularly important when GLP-1 receptor agonists are considered for patients with obesity-related KOA, because this population often includes older adults, patients with multimorbidity, and individuals approaching elective arthroplasty. Gastrointestinal adverse events, including nausea, vomiting, diarrhea, constipation, and reduced oral intake, may limit dose escalation and lead to treatment interruption or discontinuation. In older or frail patients, these symptoms may also increase the risk of dehydration, insufficient protein intake, and functional decline. Therefore, GLP-1 receptor agonists should not be prescribed as isolated weight-loss agents in KOA patients, but should be embedded within a monitored care plan that includes nutritional assessment, hydration advice, and follow-up of tolerability.

Body-composition change is another practical concern. Substantial weight loss may reduce knee loading and improve symptoms, but clinically relevant loss of lean mass may worsen sarcopenia, quadriceps weakness, balance, walking capacity, and postoperative recovery. This issue is especially relevant in patients with pain-limited mobility, older age, or advanced KOA. For this reason, weight reduction should be accompanied by resistance exercise, adequate protein intake, and functional monitoring whenever possible. Treatment success should therefore be judged not only by body-weight reduction, but also by preservation of muscle strength, mobility, and rehabilitation capacity (Pantazopoulos et al., 2025).

Perioperative management also requires careful coordination. GLP-1 receptor agonists may delay gastric emptying, raising concerns about retained gastric contents and aspiration risk during anesthesia. Patients being optimized for total knee arthroplasty should have GLP-1 receptor agonist use reviewed jointly by the prescribing clinician, anesthesiology team, and arthroplasty surgeon. Current perioperative decisions should be individualized according to drug type, dose-escalation phase, gastrointestinal symptoms, aspiration risk, and surgical urgency, rather than managed by a single universal rule (Oprea et al., 2024; Kindel et al., 2025).

Long-term use and discontinuation also need to be considered. In the STEP 1 extension study, withdrawal of semaglutide was followed by substantial weight regain and partial reversal of cardiometabolic improvements, suggesting that short-term pharmacologic weight loss may not be durable without a maintenance strategy (Wilding et al., 2022). Although this evidence was not generated in a KOA population, it is highly relevant to obesity-related KOA because recurrence of weight gain may reduce the durability of pain relief, rehabilitation gains, and surgical optimization. Cost-related nonadherence may further limit sustained treatment, particularly when insurance coverage is incomplete, long-term therapy is unaffordable, or medication supply is unstable. This issue is especially important in obesity-related KOA because treatment benefit may depend on continuous weight management rather than short-term drug exposure. If GLP-1 receptor agonists are accessible only to selected patients with stronger financial resources or better insurance coverage, their use may widen rather than reduce disparities in musculoskeletal care. Therefore, affordability, reimbursement, access, patient preference, and expected adherence should be considered before treatment initiation, alongside clinical eligibility and safety.

Finally, clinicians should remain attentive to less common but clinically important safety concerns, including gallbladder disease and pancreatitis-related warnings. These risks should be discussed before treatment initiation, especially in patients with relevant prior history or new abdominal symptoms during therapy. Overall, GLP-1 receptor agonists may be useful for selected patients with obesity-related KOA, but their use requires careful patient selection, dose titration, adverse-event monitoring, nutritional and functional support, perioperative coordination, and realistic planning for long-term adherence.

## 7 Unresolved questions in the GLP-1 era

### 7.1 Durability of benefit and the problem of treatment discontinuation

A major unresolved question is whether the symptomatic and functional gains associated with GLP-1 receptor agonists in obesity-related KOA can be maintained over the long term. Evidence from obesity trials suggests that discontinuation of semaglutide may be followed by substantial weight regain and partial reversal of metabolic improvements (Wilding et al., 2022). However, it remains unclear whether similar patterns would translate into loss of pain relief, reduced walking tolerance, poorer rehabilitation adherence, or renewed progression toward procedural escalation in KOA populations. Future studies should therefore evaluate not only short-term changes in weight and pain, but also maintenance of benefit after dose stabilization, treatment interruption, or discontinuation.

### 7.2 Weight loss is not the only body-composition outcome that matters

Although preservation of lean mass is clinically important during GLP-1 receptor agonist therapy, the optimal strategy for monitoring and protecting muscle health in KOA populations remains uncertain. This issue is particularly relevant because quadriceps strength, balance, and walking capacity are central determinants of disability and recovery in KOA. Future trials should therefore include body-composition assessment, muscle strength testing, physical performance measures, and rehabilitation adherence as core outcomes, rather than focusing only on body weight and pain (Pantazopoulos et al., 2025).

### 7.3 Perioperative management before arthroplasty

Although current perioperative guidance emphasizes individualized assessment of GLP-1 receptor agonist use, KOA-specific perioperative pathways have not yet been established (Oprea et al., 2024; Kindel et al., 2025). Future studies should clarify how these agents should be managed before elective arthroplasty, whether perioperative interruption affects weight or glycemic stability, and whether coordinated medication management can reduce aspiration concerns without undermining preoperative optimization.

### 7.4 Structural disease modification remains unproven in humans

Perhaps the most important scientific limitation is that symptomatic improvement should not yet be interpreted as proof of structural disease modification. Weight loss has long been hypothesized to influence the structural progression of OA, but the imaging literature remains mixed. In a systematic review of weight loss and imaging outcomes in overweight or obese individuals with hip or knee OA, Daugaard and colleagues concluded that there was no consistent evidence that weight loss altered structural OA pathology, while also emphasizing major heterogeneity in the structural endpoints used across studies (Daugaard et al., 2020). More specifically for GLP-1 therapy, a 2025 systematic review of preclinical and human studies found encouraging signals of structural, immunomodulatory, and analgesic benefit, but also noted that human evidence remains limited, with only a small number of clinical studies and very sparse structural data (Cheng et al., 2025). Therefore, while the current evidence supports GLP-1-based therapy as a clinically meaningful strategy for obesity-related symptom control and pathway optimization, it does not yet justify the stronger claim that these agents are disease-modifying treatments for KOA in the human setting (Daugaard et al., 2020; Cheng et al., 2025). Future trials will need to incorporate imaging endpoints, cartilage and bone biomarkers, body-composition assessment, and longer follow-up if the field is to move from symptomatic pathway redesign toward true structure-informed precision care.

## 8 Future directions

### 8.1 Trials must move beyond pain endpoints toward pathway-relevant outcomes

The next-generation of studies in obesity-related KOA should not be limited to short-term symptom endpoints. STEP 9 established that semaglutide can improve body weight and knee pain in people with obesity and KOA, and the TRIUMPH program indicates that newer incretin-based therapies are now being formally developed for obesity-related complications including knee osteoarthritis (Bliddal et al., 2024; Giblin et al., 2026). However, a high-impact research agenda now requires broader outcomes, including durability of benefit after long-term treatment, body-composition changes during weight loss, preservation of muscle function, reduction in analgesic or injection use, time to arthroplasty, perioperative optimization, and structural imaging progression (Wilding et al., 2022; Pantazopoulos et al., 2025; Cheng et al., 2025). This expansion is especially important because emerging observational evidence has already shown that GLP-1 receptor agonist exposure may be associated not only with altered symptom trajectories, but also with differences in major joint injections and conversion to arthroplasty in patients with preexisting OA, underscoring the need for future trials to capture pathway-level outcomes rather than pain alone (Porto et al., 2025).

### 8.2 Real-world and surgical-transition evidence now deserve priority

A second priority is to define how these therapies perform in the transition zone between medical management and surgery. This is no longer a theoretical issue. A recent systematic review found that current evidence suggests GLP-1 receptor agonists may improve total joint arthroplasty outcomes, particularly by reducing infection risk and readmission, although the underlying studies remain heterogeneous and largely retrospective (Lee V. et al., 2025). A subsequent systematic review and meta-analysis of 346,899 patients further reported lower 90-day periprosthetic joint infection after TKA, lower 90-day revision after THA, and reduced readmission rates following THA and TKA among GLP-1RA users, while not showing a significant increase in aspiration or pneumonia, although a signal for myocardial infarction after TKA was noted (Lee S. et al., 2025). These findings suggest that future KOA studies should no longer evaluate anti-obesity pharmacotherapy in isolation. Instead, future studies should examine whether GLP-1RA therapy is independently associated with improved surgical readiness, reduced perioperative risk, delayed need for arthroplasty, or differential benefit across patient subgroups, using designs capable of minimizing confounding by indication and healthier-user bias (Porto et al., 2025; Lee V. et al., 2025; Lee S. et al., 2025).

### 8.3 Implementation, eligibility, and affordability will determine real-world impact

Even if efficacy continues to strengthen, implementation will remain a major determinant of clinical impact. Economic modeling in patients with obesity and knee osteoarthritis suggests that both semaglutide and tirzepatide would generally be considered cost-effective compared with usual care, with tirzepatide offering the more favorable return on investment once willingness-to-pay thresholds exceed $57,400 per QALY (Betensky et al., 2025). However, economic evaluation should also compare GLP-1 receptor agonists with other obesity-treatment strategies rather than with usual care alone. Lifestyle intervention, multidisciplinary weight-management programs, bariatric surgery, and other anti-obesity medications differ in cost, durability, accessibility, tolerability, and expected weight-loss magnitude. Therefore, the practical value of GLP-1 receptor agonists in obesity-related KOA will depend not only on efficacy, but also on whether they provide sustainable benefit relative to available alternatives within a given healthcare system. At the same time, real-world evidence indicates that weight loss achieved in routine practice is often lower than that seen in randomized trials overall, although outcomes approach trial-level results in highly adherent patients; gastrointestinal adverse effects remain a common reason for discontinuation (Thomsen et al., 2025). These implementation realities align with recent obesity guidelines. The 2025 clinical practice guideline update for adult obesity management identified pharmacotherapy as a key component of comprehensive obesity care, recommended that initiation be guided not only by BMI but also by central adiposity and adiposity-related complications, and specifically included osteoarthritis among the relevant complication subgroups (Pedersen et al., 2025). Similarly, the 2026 WHO guideline recognized obesity as a chronic, relapsing disease requiring long-term care and emphasized that implementation of GLP-1-based treatment depends on affordable access, health-system readiness, and integrated person-centered care rather than medication alone (Celletti et al., 2026). Consistent with this cautious implementation perspective, a recent systematic review and meta-analysis focusing specifically on GLP-1 receptor agonists in knee osteoarthritis also suggested potential benefits for weight-related outcomes while emphasizing that the available KOA-specific evidence remains limited and that further trials are needed to clarify efficacy, safety, and durability (Singh et al., 2025). Accordingly, the future of GLP-1-based treatment in obesity-related KOA will depend not only on efficacy, but also on whether health systems can build multidisciplinary pathways that connect obesity medicine, rehabilitation, musculoskeletal care, and arthroplasty services around shared clinical goals.

## 9 Conclusion

GLP-1 receptor agonists have expanded the therapeutic discussion in obesity-related knee osteoarthritis by making substantial pharmacologic weight loss achievable for selected patients. At present, their most defensible role is not as proven structure-modifying therapy for KOA, but as a potential component of integrated obesity-directed care that may improve pain, function, rehabilitation participation, and perioperative optimization. Direct cartilage-, synovium-, or bone-modifying effects remain insufficiently established in humans, and the available evidence is still limited by sparse KOA-specific trials, short-to-medium follow-up, limited structural endpoints, and uncertainty regarding long-term safety, adherence, affordability, and surgical outcomes. Future studies should determine whether GLP-1-based therapy can produce durable, clinically meaningful, and cost-effective benefits within multidisciplinary KOA care pathways.
